# Antibiotic Treatment Practices and Microbial Profile in Diabetic Foot Ulcers: A Retrospective Cohort Study

**DOI:** 10.7759/cureus.67084

**Published:** 2024-08-17

**Authors:** Charles P Daniel, Kevin M Sittig, Maxwell J Wagner, Collins Cade, Wendy Chriss

**Affiliations:** 1 School of Medicine, Louisiana State University Health Sciences Center, Shreveport, USA; 2 Department of Surgery, Louisiana State University Health Sciences Center, Shreveport, USA

**Keywords:** underserved patient population, atypical pathogen, antibiotic susceptibility, : acute kidney injury, vancomycin nephrotoxicity, antibiotic policy, diabetes-related complications, diabetic foot infection, diabetic foot ulcers management, “diabetes mellitus”

## Abstract

Aim and objective

Diabetic foot ulcers (DFUs) are a frequent complication of diabetes mellitus, impacting more than one in 10 diabetic patients, with roughly half of these ulcers progressing to infection. Existing literature indicates that these infections are predominantly polymicrobial, with gram-positive isolates being the most common. This microbial profile informs the empiric antibiotic strategies employed in first-world countries, often including highly potent nephrotoxic antibiotics. This retrospective cohort study aims to assess the microbial profile and antibiotic treatment practices in patients with infected DFUs at Ochsner LSU Health Shreveport Academic Medical Center in Shreveport, Louisiana, United States.

Materials and methods

A total of 115 patients diagnosed with infected DFUs were included in the study. Patient records were reviewed to identify bacterial pathogens cultured from foot wounds, antibiotic treatment regimens administered, and the prevalence of acute kidney injury (AKI).

Results

The study found a predominance of gram-negative isolates (199; 59.4%), facultative anaerobes (246; 73.4%), and polymicrobial infections (67; 78.8%) in infected DFUs. Vancomycin was administered to 95 patients (82.6%), with only a small number subsequently testing positive for methicillin-resistant *Staphylococcus aureus* (MRSA). Combination therapy with vancomycin and Zosyn was given to 71 patients (61.7%), which increased the potential risk of antibiotic-induced nephrotoxicity. AKI was prevalent, affecting 58 patients (50.4%).

Conclusions

This study highlights a discrepancy between the microbial profile of infected DFUs and empiric antibiotic treatment practices at Ochsner LSU Health Shreveport Academic Medical Center. The predominance of gram-negative bacteria underscores the need for a polymicrobial, gram-negative-focused empiric treatment approach. Alternative antibiotics with broad-spectrum coverage and minimal nephrotoxicity, such as ceftriaxone, clindamycin, metronidazole, amoxicillin-clavulanate, and linezolid, should be considered. Tailored antibiotic strategies, guided by local microbial profiles and patient-specific factors, are essential to optimize treatment outcomes in this high-risk population.

## Introduction

Diabetes mellitus affects approximately 382 million individuals worldwide [1]. Data indicates that diabetic foot ulcers (DFUs) are present in about 57 million (15%) of diabetics, with approximately 22.9 million (40%) of these ulcers progressing to infection [2]. Unfortunately, outcomes for patients with infected DFUs are generally unfavorable, with a significant proportion undergoing lower limb amputation [3,4]. While gram-positive isolates dominate cultures in high-income countries, gram-negative isolates are more prevalent in lower-income countries [1,5]. Current treatment guidelines recommend a gram-positive-focused empiric antibiotic approach. However, patients with DFUs often present with delayed clinical manifestations due to comorbidities such as peripheral vascular disease, diabetic neuropathy, and poor metabolic control [6-9].

Research has indicated a need to better anticipate wound pathogen profiles for direct treatment with pathogen-sensitive antibiotics [10]. Although wound cultures provide valuable information, their turnaround time, averaging one to three days, necessitates an empiric antibiotic approach while awaiting results [11]. Furthermore, bacterial culture prevalence correlates with geographic region, economic status, and the bioburden present at clinical manifestation. Large-scale studies suggest that DFUs in high-income countries are predominantly populated by multidrug-resistant, gram-positive isolates, but these studies may overlook the unique clinical profile of local and regional populations, including demographic disparities such as poverty and homelessness [1,8,12-14]. Given these considerations, there is a pressing need to update empirical antibiotic therapy policies to ensure appropriate coverage and sensitivity to the pathogen profiles specific to regions with lower socioeconomic status, such as Shreveport, Louisiana, United States [8,14-30].

## Materials and methods

Study design

The study design is a retrospective cohort study that analyzed 115 patients treated at LSU Health Shreveport for diabetic foot infection (code: E11.628 L08.9) between June 2011 and June 2023. The study received institutional review board approval, and pertinent data were collected through a review of patient electronic medical records.

Inclusion and exclusion criteria

Inclusion criteria required evidence of a prior diagnosis of diabetes mellitus, the presence of a diabetic foot wound, and the completion of a wound tissue and/or swab culture from that diabetic foot wound assessing for specific bacterial pathogens. Patients were excluded if they did not complete a wound culture or if the culture could not be processed, resulting in an inconclusive report.

Data collection

Patient charts were reviewed for bacterial pathogen type, antibiotic therapy exposure, and prevalence of acute kidney injury (AKI). Each variable was counted once per patient to ensure accurate population biodiversity profiling. Patient demographics such as date of birth, gender, and BMI were recorded. Additional demographic and socioeconomic data were obtained from the United States Census Bureau.

Statistical analysis

Statistical data analysis was conducted to ascertain the predominant bacterial pathogens in infected DFUs among patients at LSU Health Shreveport. The study aimed to characterize the wound bioburden and biodiversity, evaluate pharmacologic exposure to nephrotoxic antimicrobial drugs, and determine the prevalence of AKI. The findings were intended to direct evidence-based patient antibiotic management and enhance treatment outcomes for the local patient population.

## Results

Pathogen profile

**Figure 1:**
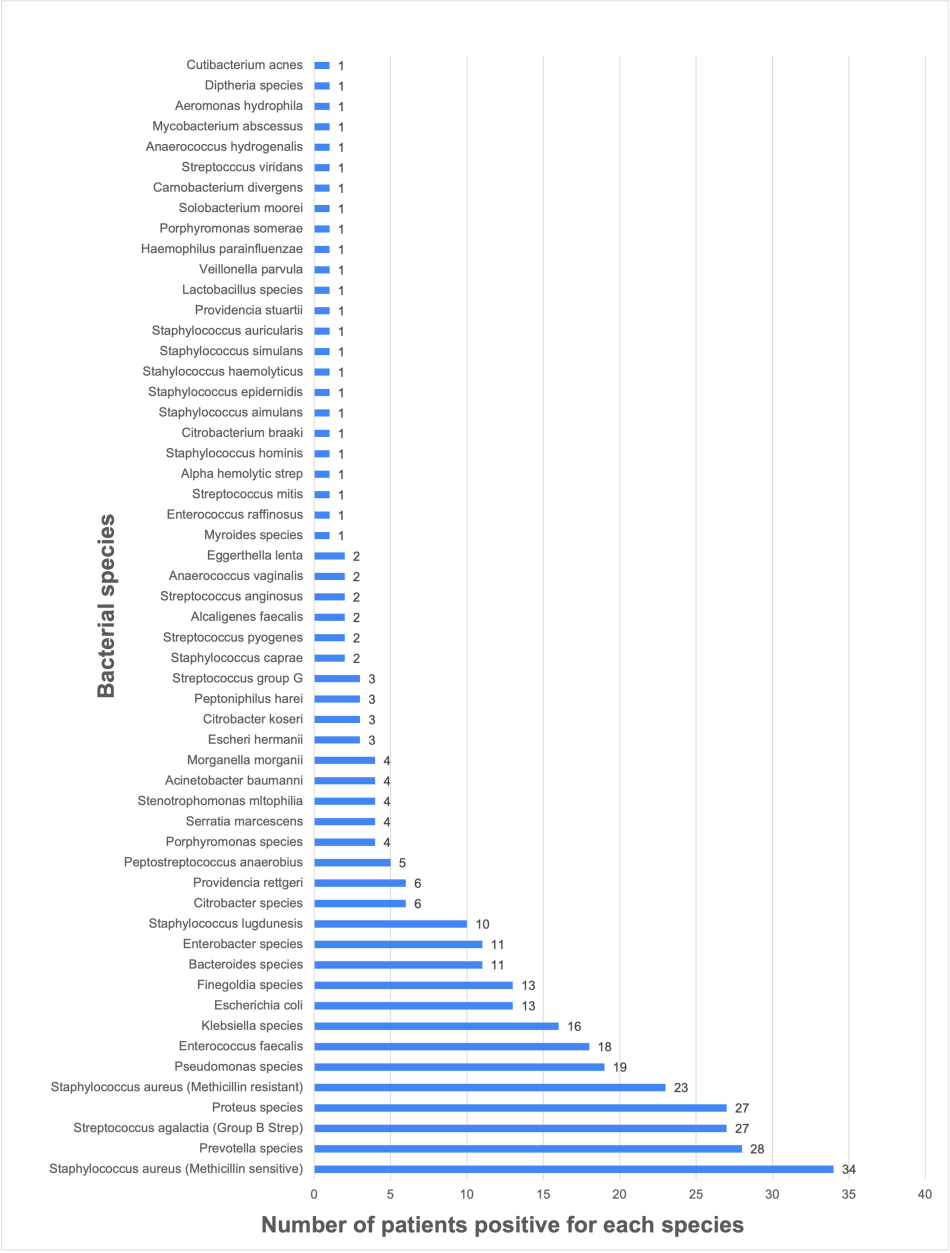
Cultured diabetic foot wound pathogens The y-axis of the figure represents the confirmed bacterial isolates identified from swab cultures taken from the patients’ infected diabetic foot wounds at the time of their clinical presentation. The x-axis shows the number of patients with each identified bacterial isolate.

The study included 115 patients treated at LSU Health Shreveport for infected DFUs. Of the 115 patients, 84 (73.0%) wound cultures identified bacterial isolates. The patient population demonstrated a high level of biodiversity, with an average of 2.94 distinct bacterial pathogens per patient and up to 18 pathogens identified in a single wound. Most patients presented with polymicrobial foot infections (67 patients). Methicillin-sensitive *Staphylococcus aureus *(MSSA) found in 34 patients was the most commonly cultured bacterial isolate, followed by *Prevotella *species in 28 patients, *Streptococcus agalactiae (*Group B Streptococcus) in 27 patients, and *Proteus *species in 27 patients. Among the 335 identified bacterial pathogens spanning 56 species, 199 (59.4%) were gram-negative isolates, while 136 (40.6%) were gram-positive. Facultative anaerobes were the most prevalent wound culture isolates, with 246 identified pathogens, followed by anaerobes with 76 identified pathogens and 13 aerobic pathogens (Figure 1).

Antibiotic exposure profile

Among the 115 patients studied, vancomycin was the most frequently administered antimicrobial agent (95 patients), followed by piperacillin-tazobactam (Zosyn) (73 patients). Cefazolin was administered to 60 patients, while amoxicillin-clavulanate and cefepime were administered to 56 and 45 patients, respectively. Among the 95 patients who received vancomycin, only 23 subsequently had a methicillin-resistant S*. aureus *(MRSA)-positive wound culture. The most common dual antimicrobial therapy was vancomycin and Zosyn (71 patients), followed by vancomycin and cefazolin (56 patients).

Comorbidity profile

Among the 115 patients studied, 58 had one or more episodes of AKI. Within this AKI group, 51 patients received vancomycin, and 40 patients received both vancomycin and Zosyn. Additionally, seven of the 51 patients who received vancomycin and had AKI did not produce a gram-positive bacterial culture isolate. Among the 71 patients treated with vancomycin and Zosyn, 40 experienced an AKI, while 34 of the 56 patients treated with vancomycin and cefazolin also exhibited an AKI. In total, AKI was found in 41 patients who were administered Zosyn, 35 patients who received cefazolin, 31 patients who were prescribed Augmentin, and 29 patients who were given cefepime.

Other study population data

In the study population, the average age at diagnosis of an infected DFU is 59 years. The average BMI is 30.6 kg/m², indicating a classification as medically obese. A majority of the population is male (76 patients), with 39 females. All patients received treatment at Ochsner LSU Health Shreveport Academic Medical Center.

## Discussion

The study encompassing 115 patients unveiled a significant predominance of gram-negative isolates 199 (59.4%), facultative anaerobes 246 (73.4%), and polymicrobial infections 67 (78.8%), indicative of a distinct bacterial bioburden compared to high-income Western countries where gram-positive isolates typically prevail. Despite this, a striking 95 (82.6%) patients received vancomycin, primarily targeting gram-positive bacteria, with only a minority subsequently testing positive for MRSA. Moreover, the administration of vancomycin combined with Zosyn to 71 (61.7%) patients substantially escalates the risk of antibiotic-induced nephrotoxicity. Notably, AKI was present in 58 (50.4%) patients.

These findings underscore that the bacterial pathogen profile in diabetic foot wounds at our study center mirrors low-income non-Western countries, reflecting local poverty rates and unique infection risk factors. Empiric antibiotic practices extrapolated from Western data inadequately address this population’s bacterial profile. Vancomycin fails to cover the majority of cultured pathogens, necessitating a shift toward a polymicrobial, gram-negative-focused empiric treatment approach. Such an approach should target prevalent bacteria like MSSA, *Prevotella *species, *Proteus *species, and *S. agalactiae*, tailored post-culture to minimize the use of vancomycin while ensuring sensitivity to cultured pathogens.

Recently, the empiric antibiotic treatment approach for infected DFUs at LSU Health Shreveport shifted from a combination of vancomycin and piperacillin-tazobactam (Zosyn) to include vancomycin, cefepime, and occasionally metronidazole (Flagyl). Vancomycin primarily targets gram-positive bacteria, particularly MRSA, while piperacillin-tazobactam (Zosyn) is a broad-spectrum antibiotic effective against both gram-positive and gram-negative bacteria. Cefepime, a fourth-generation cephalosporin, offers extended-spectrum coverage against gram-positive and gram-negative bacteria, while metronidazole (Flagyl) addresses anaerobic bacterial infections. Alternative antibiotic therapies for this population include amoxicillin-clavulanate (Augmentin), which has broad-spectrum coverage, and cefazolin, a first-generation cephalosporin effective against gram-positive bacteria with limited gram-negative activity [5,6,10,20-22].

Potentially less nephrotoxic alternative therapies include ceftriaxone, clindamycin, metronidazole, amoxicillin-clavulanate, and linezolid. Ceftriaxone, a third-generation cephalosporin, and clindamycin, a lincosamide antibiotic, offer broad-spectrum coverage against both gram-positive and gram-negative bacteria, including *S. aureus *and *S. agalactiae*, with minimal nephrotoxicity. Metronidazole provides coverage against anaerobic bacteria, including *Prevotella*, and can be used as part of a combination antibiotic regimen with minimal nephrotoxicity. Amoxicillin-clavulanate, another broad-spectrum antimicrobial agent, and linezolid, with activity against gram-positive bacteria including MRSA, present lower risks of nephrotoxicity compared to vancomycin.

The choice of antibiotic therapy should carefully consider the patient’s individual factors, including allergies, renal function, and local antimicrobial resistance patterns. Consulting with an infectious disease specialist or adhering to local antimicrobial stewardship guidelines can facilitate the optimization of antibiotic selection while minimizing nephrotoxicity.

Considerations for antibiotic choice in diabetic foot infections must encompass both pathogen sensitivity coverage and patient comorbidities. Diabetic patients face a significantly elevated risk of AKI compared to nondiabetic individuals, with data indicating an eightfold higher incidence of AKI in diabetic patients [23-26]. Diabetes is also a standalone risk factor for AKI recurrence. Among published studies, AKI occurs in roughly 10-20% of diabetic patients, with the range highly dependent on both medical intervention and the region of treatment [15-17,27]. Consequently, clinicians evaluating antibiotic therapy for diabetic foot infections must prioritize the increased susceptibility of this patient population to kidney injury. Moreover, there exists a concerning trend in current vancomycin drug dosing practices, with a loading dose to achieve supra-therapeutic levels, despite evidence indicating an increased risk of AKI, especially in the high-risk diabetic population. Vancomycin has demonstrated a risk of AKI that exceeds twofold when compared to a control population [18-22]. Piperacillin-tazobactam (Zosyn) also exhibits an increased risk of antibiotic-associated AKI, which escalates in a dose-dependent manner when used alone. Additionally, the combination of vancomycin and piperacillin-tazobactam (Zosyn) amplifies the incidence of drug-associated AKI by two to three times that of vancomycin alone. Similarly, amoxicillin-clavulanate (Augmentin) has demonstrated a dose-dependent increase in AKI risk, akin to penicillin-tazobactam [5,21,28]. While cefazolin and cefepime have been linked to rare cases of acute tubular necrosis and acute interstitial nephritis, cephalosporins generally present a lower-risk option among broad-spectrum antibiotics [10,21,28].

The choice of antibiotic therapy should carefully consider the patient’s individual factors, including allergies, renal function, and local antimicrobial resistance patterns. Consulting with an infectious disease specialist or adhering to local antimicrobial stewardship guidelines can facilitate the optimization of antibiotic selection while minimizing nephrotoxicity.

This study examined patients treated at Ochsner LSU Health Shreveport Academic Medical Center, a level I trauma center and state-funded healthcare facility in northwest Louisiana. The facility predominantly serves local patients, with 48.3% of inpatients coming from three nearby zip codes (71109, 71106, and 71107) in Caddo Parish. Caddo Parish, with a population of 229,025, has significant socioeconomic challenges, with a poverty rate of 23.6%, a median household income of $43,153, and a per capita income of $29,220. These figures contrast sharply with national averages, highlighting the increased vulnerability of the local population to infections with nontraditional wound pathogens. Consequently, unique considerations are warranted in patient care for this distinct population [29,30].

When comparing our findings to existing studies, our results align with research from low-income, non-Western countries, which also report a predominance of gram-negative and polymicrobial infections in DFUs. For instance, studies from India and sub-Saharan Africa have shown similar microbial profiles, highlighting the significant regional differences in pathogen prevalence and the limitations of applying Western antibiotic protocols universally. Additionally, our study corroborates previous findings that underscore the nephrotoxicity risks associated with common empiric therapies like vancomycin and Zosyn, emphasizing the need for tailored antibiotic strategies. These comparisons further validate our conclusion that a more nuanced, region-specific approach to antibiotic therapy is crucial for improving patient outcomes in diverse healthcare settings.

Limitations

This study has several limitations that should be considered when interpreting the results. Firstly, as a retrospective cohort study, it relies on the accuracy and completeness of patient records, which can be subject to documentation errors and missing data. This limitation may affect the reliability of the findings related to bacterial pathogen identification, antibiotic treatment regimens, and the incidence of AKI. Secondly, the study was conducted at a single academic medical center, Ochsner LSU Health Shreveport, which may limit the generalizability of the results to other settings or populations. The microbial profiles and antibiotic practices observed may be specific to the local patient population and healthcare practices and may not be representative of other regions or institutions. Thirdly, the study focused on a relatively small sample size of 115 patients, which may not capture the full diversity of microbial pathogens and treatment responses in the broader DFU population. A larger, multicenter study would be necessary to validate these findings and provide more robust evidence for clinical practice. Furthermore, the reliance on wound culture data to identify bacterial pathogens may not fully capture the complexity of polymicrobial infections. Some pathogens may not be detected due to limitations in culture techniques, and molecular methods could provide a more comprehensive understanding of the microbial environment in DFUs. Lastly, the study did not account for potential confounding factors such as patient comorbidities, variations in wound care practices, or differences in antibiotic stewardship policies, which could influence the outcomes observed. Future research should aim to include these variables to better understand their impact on treatment efficacy and patient outcomes.

## Conclusions

These study findings underscore that the bacterial pathogen profile in diabetic foot wounds at our study center mirrors that of low-income non-Western countries, reflecting local poverty rates and unique infection risk factors. Empiric antibiotic practices extrapolated from Western data inadequately address this population’s bacterial profile, necessitating a shift toward a polymicrobial, gram-negative-focused empiric treatment approach. The choice of antibiotic therapy should carefully consider the patient’s individual factors, especially renal function and local antimicrobial resistance patterns.

In summary, the study highlights the need for tailored antibiotic strategies that address the specific microbial landscape of DFUs in our patient population. A shift toward a more targeted, gram-negative-focused empiric treatment approach, alongside careful consideration of nephrotoxicity risks, can improve patient outcomes and better manage the complex infections associated with DFUs.
